# Assessing Telemedicine Efficiency in Follow-up Care With Video Consultations for Patients in Orthopedic and Trauma Surgery in Germany: Randomized Controlled Trial

**DOI:** 10.2196/36996

**Published:** 2022-07-27

**Authors:** Jennifer Muschol, Martin Heinrich, Christian Heiss, Gero Knapp, Holger Repp, Henning Schneider, Ulrich Thormann, Johanna Uhlar, Kai Unzeitig, Christian Gissel

**Affiliations:** 1 Department of Health Economics Justus Liebig University Giessen Giessen Germany; 2 Department of Trauma, Hand and Reconstructive Surgery University Hospital Giessen Giessen Germany; 3 Institute of Medical Informatics Justus Liebig University Giessen Giessen Germany

**Keywords:** telemedicine, video consultations, follow-up, efficiency, orthopedic, trauma surgery, mobile phone

## Abstract

**Background:**

Telemedicine can help mitigate important health care challenges, such as demographic changes and the current COVID-19 pandemic, in high-income countries such as Germany. It gives physicians and patients the opportunity to interact via video consultations, regardless of their location, thus offering cost and time savings for both sides.

**Objective:**

We aimed to investigate whether telemedicine can be implemented efficiently in the follow-up care for patients in orthopedic and trauma surgery, with respect to patient satisfaction, physician satisfaction, and quality of care.

**Methods:**

We conducted a prospective randomized controlled trial in a German university hospital and enrolled 60 patients with different knee and shoulder conditions. For follow-up appointments, patients received either an in-person consultation in the clinic (control group) or a video consultation with their physician (telemedicine group). Patients’ and physicians’ subsequent evaluations of these follow-up appointments were collected and assessed using separate questionnaires.

**Results:**

On the basis of data from 52 consultations after 8 withdrawals, it was found that patients were slightly more satisfied with video consultations (mean 1.58, SD 0.643) than with in-clinic consultations (mean 1.64, SD 0.569), although the difference was not statistically significant (*P*=.69). After excluding video consultations marred by technical problems, no significant difference was found in physician satisfaction between the groups (mean 1.47, SD 0.516 vs mean 1.32, SD 0.557; *P*=.31). Further analysis indicated that telemedicine can be applied to broader groups of patients and that patients who have prior experience with telemedicine are more willing to use telemedicine for follow-up care.

**Conclusions:**

Telemedicine can be an alternative and efficient form of follow-up care for patients in orthopedic and trauma surgery in Germany, and it has no significant disadvantages compared with in-person consultations in the clinic.

**Trial Registration:**

German Clinical Trials Register DRKS00023445; https://www.drks.de/drks_web/navigate.do?navigationId=trial.HTML&TRIAL_ID=DRKS00023445

## Introduction

### Background

International health care systems are facing several major challenges. Some of these challenges are structural and have evolved over the years, while others have occurred as sudden shocks. Demographic change and a shortage of health care professionals are among the increasingly important structural challenges and have been impacting patient care for years. On the one hand, the rising number of older and multimorbid patients is leading to a demographic change, which is associated with a higher demand for health care services. On the other hand, there is a growing shortage of specialists to meet this demand efficiently. Simultaneously, an asymmetrical distribution of medical service providers leads to deficits in health care. Particularly in rural regions, patients have to travel longer distances, and thus incur higher costs. In the long term, this could lead to limited access to health care for a subset of patients [1-3].

Beyond these structural challenges, health care systems have recently had to cope with the sudden COVID-19 pandemic [4], starting with its global outbreak in March 2020 [5]. The pandemic has placed several important restrictions on the delivery of medical care; for example, social distancing has become necessary to avoid infections [6,7]. Hospitals, which are at a particular risk of causing a pandemic outbreak owing to their high number of interactions and patients, have introduced protective measures [8,9]. Some patients avoid medical appointments for fear of infection [10,11], and hospitals have been postponing nonurgent treatments and interventions to save resources [6,7,12]. The lack of physician-patient interactions and avoidance of treatments could lead to worsening health outcomes in the future [13].

Both structural challenges and the pandemic shock will likely have a long-term impact on the health care system and delivery of care. As a result, existing structures will need to be reconsidered [2,3,14].

One important tool for overcoming these challenges and guaranteeing effective health care in the medium to long term could be the use of telemedicine. Telemedicine offers the ability to provide medical care through real-time video consultations, without the need for personal contact and regardless of location. This could free up clinical resources, improve access to care, and increase safety for patients and medical staff [3,15-18].

Telemedicine is already being applied successfully in various medical fields [19,20], but its use has so far been less common in orthopedic and trauma surgery owing to the specialty’s heavy reliance on palpation and dynamic testing and telemedicine’s inherent constraints [7]. In addition, regardless of medical specialty, there were barriers that still negatively impacted readiness for adoption despite the benefits of telemedicine. These barriers included, for example, resistance to change, lack of technical literacy, or uncertainties about costs and reimbursement [21,22]. However, since the outbreak of the COVID-19 pandemic, the need for telemedicine has increased considerably in the field of orthopedic and trauma surgery, among other medical areas [7,16,18]. More specifically, telemedicine can support outpatient care in hospitals, such as follow-up examinations to prior interventions [23]. While vital for successful treatment [24], these follow-up examinations entail a travel burden for patients who are often immobile or in pain due to their condition. Therefore, telemedicine could offer a suitable alternative [25].

In 2019, German hospitals admitted a total of 854,410 patients in orthopedic surgery and 759,356 patients in trauma surgery, making the combination of orthopedic and trauma surgery one of the largest areas of care in Germany [26]. Increasing the use of telemedicine to relieve clinics and patients of unnecessary burdens in this broad field could provide significant benefits. Although these benefits can be determined only by clinical evaluation, randomized controlled trials (RCTs) examining telemedicine in orthopedic and trauma surgery are rare, with few exceptions.

In an RCT, Buvik et al [23,27,28] compared standard consultations in an orthopedic outpatient clinic of a hospital with video consultations assisted by a trained nurse at a regional medical center in Norway. It was shown that telemedicine is a safe alternative, that its use can be cost-effective, and that there are no significant differences in patient satisfaction and health status between the treatment group and the control group [23,27,28].

Sathiyakumar et al [29] also found no significant differences in patient satisfaction between telemedicine and in-person follow-up for patients with closed orthopedic trauma injuries in a level 1 trauma center in the United States. In this RCT, telemedicine was associated with time and travel savings for patients [29].

The use of telemedicine for a postoperative follow-up of arthroscopic rotator cuff repair surgery was investigated by Kane et al [30] in the United States. Their RCT concluded that telemedicine can be used safely and effectively for this condition, that patient satisfaction was similar, and that time savings were achieved for both patients and physicians [30].

### Objectives

However, prior research has left several questions unaddressed, which are considered based on our research design. One of them is whether the use of telemedicine is efficient not only for a restricted number of individual diseases but also for a wider range of medical conditions. Another important question is with regard to the practicality of telemedicine without the need to involve additional staff to assist patients during video consultations [23,27,28]. Furthermore, it is questionable whether international study results can be transferred to the German health care system, especially because studies show that German patients are skeptical about the use of telemedicine [31].

The aim of our RCT was to investigate whether telemedicine can be used efficiently in follow-up care for patients in orthopedic and trauma surgery in Germany. To answer this question, the RCT compared an in-person consultation in a German university hospital (level 1 trauma center) with the use of telemedicine, namely a video consultation between the physician and patient. All consultations were for the follow-up care of patients with knee and shoulder conditions who displayed a variety of conditions, had previously been treated in the clinic, and were eligible to participate in the study. For their video consultation, patients did not have to travel to the clinic but could have their follow-up appointment on the web, regardless of their location. For this study, the aspects of patient satisfaction, physician satisfaction, and quality of care were considered as the most important factors to quantify the output of telemedicine. Therefore, they were included in the evaluation of telemedicine under the overarching term “efficiency.” It is hoped that studying telemedicine in broad-based use for follow-up care and analyzing its effects comprehensively will contribute to informing health care providers’ decision-making in future.

## Methods

### Study Design

This study was conducted as an open, prospective, interventional, 1:1 randomized controlled monocenter trial at a German university hospital (University Hospital Giessen, Department of Trauma, Hand and Reconstructive Surgery). The randomized and controlled design is based on the CONSORT (Consolidated Standards of Reporting Trials) [32]. The effects of telemedicine on follow-up care were examined with the parallel implementation of an intervention group, which received follow-up care through a real-time video consultation, and a control group, which received a standard follow-up consultation in the clinic.

### Ethics Approval

The local ethics committee of the University of Giessen reviewed and approved this study (AZ 73/20). The study was registered in the German Clinical Trials Register (ID: DRKS00023445).

### Definition and Characteristics of the Trial Population

The trial population consisted of knee and shoulder patients who have already been treated in the department. The patients’ medical conditions varied, and Multimedia Appendix 1 lists their ICD-10 codes. Their medical conditions included, for example, fractures of the patella and femur, impingement syndrome of the shoulder, and orthopedic joint implants.

To adequately guarantee the safety of patient care, recruitment for the RCT observed the following inclusion criteria in addition to the ICD-10 codes: (1) patients need the ability to consent, as well as the mental and physical ability to participate in the telemedical consultation. (2) As part of the consultation, patients’ conditions should require no more than a visual examination and a conversation without the need to be touched or moved by the treating physician or other physical interactions. For legal reasons, (3) a previous outpatient or inpatient stay at the clinic is required, and (4) patients must be ≥18 years. To be able to use telemedicine, it is assumed that (5) patients own a computer, laptop, tablet, or smartphone, including a microphone and camera, and (6) they have a stable internet connection. Finally, (7) patients have to speak German to understand the declaration of consent.

The concern with patient safety is also reflected in the exclusion criteria. Thus, patients with (1) neurological diseases that do not allow the use of computer systems and (2) patients with a diagnosis of dementia, blindness, or deafness were excluded. In addition, patients were excluded if they (3) have a need for in-person presence and on-site diagnostics or treatments (eg, medical imaging, laboratory, stitches, or drainage) or (4) have to be touched or moved by the treating physician. This ensured that patients who required personal contact with a physician were not at risk. Finally, (5) a lack of willingness to participate in the study or (6) the failure to consent were further added to the exclusion criteria.

### Recruitment and Randomization of Study Participants

After the initial screening for inclusion and exclusion criteria, patients were asked either at the clinic or by telephone if they would like to participate in the study during their next follow-up appointment. To be able to participate, the patients had to provide informed consent after receiving written and oral information. Consent could be withdrawn at any time, without providing reasons.

We followed a 2-armed parallel group design, and patients enrolled in the study were randomly assigned in a 1:1 ratio to either the intervention arm (telemedicine follow-up) or the control arm (in-person follow-up consultation in the clinic). To ensure better balance between the arms while minimizing predictability, block randomization with randomly selected block sizes of 4, 6, and 8 was applied [32]. One member of the study staff organized the allocation of the blocks, while a different member performed the randomization of the patients. For this purpose, sealed envelopes were used. Given that the intervention was a video consultation, blinding of the physicians or patients was not possible. At the end of the recruitment process, depending on the treatment arm, the patients received an appointment either in the clinic or for a video consultation. Patients in the intervention group also received written instructions on how to be prepared for the video consultation to minimize potential technical difficulties.

### Procedures

#### Intervention Arm

Study participants who were assigned to the intervention arm received a one-time telemedical follow-up via a real-time videoconference instead of a standard consultation in the department. The one-time appointment was intended to avoid bias through learning effects. The web-based video consultation used the web-based software CLICKDOC of the German telemedicine provider CGM Mobile Services GmbH. This software is certified for and widely used in the German health care system. On the day of their appointment, the patients received log-in details for the video consultation from their physicians via SMS text message or email. No additional registration was required for the video consultation. At the arranged time, both the attending physician and the patient logged in to the software program, and the video consultation was conducted. Patients were able to use a computer, laptop, tablet, or smartphone to join the video consultation. If technical problems were noted, patients were contacted by phone and scheduled for a clinic visit, as needed. Immediately after the video consultation, the patients received log-in details via email and were asked to evaluate the consultation via web-based questionnaires.

#### Control Arm

Study participants, who were assigned to the control group, attended a standard follow-up consultation at the university hospital. This follow-up was conducted by the same physicians who also treated the intervention arm. Immediately after the consultation, patients in the control arm were asked to fill out the questionnaires at the clinic.

### Outcome and Data Collection

To answer the research questions and analyze the outcome parameters, that is, patient satisfaction, physician satisfaction, and quality of care, different questionnaires were completed by patients and physicians.

The primary outcome patient satisfaction was measured using the German questionnaire Zufriedenheit in der ambulanten Versorgung–Qualität aus Patientenperspektive of the National Association of Statutory Health Insurance Physicians in Germany [33,34]. This validated questionnaire is an appropriate way of investigating patient satisfaction in the German outpatient sector. To adequately reflect the specific conditions of this study, individual items of the questionnaire were modified and some were added. This included excluding questions that were not relevant to the purpose of our study. The additional questions addressed whether patients experienced the treatment they wanted, how much time the physician had provided, how comfortable patients felt with the treatment, whether they were agitated, and how punctual the treatment appointment was. In addition, patients were asked to rate their overall satisfaction with their respective follow-up appointment using German school grades, where grade 1 represents “very good” and grade 6 represents “inadequate.” The other questions were answered using a 4-point Likert scale, where higher scores represented higher satisfaction. Finally, the patients were asked which option they would choose for their next follow-up appointment.

Physician satisfaction, as one of the secondary outcome parameters, was assessed by questionnaires that the physicians answered following each patient consultation. The questionnaires were self-designed and differed slightly depending on the study arm. In both groups, the physicians were asked whether all medical questions could be clarified, which option the physicians would choose for the next follow-up appointment, and how satisfied they were with the consultation. Satisfaction was also surveyed using school grades in this case. The questionnaire in the intervention group was supplemented with the questions of whether a technical irregularity occurred during the treatment and whether the consultation had to be terminated due to this malfunction.

To evaluate the quality of care as a secondary outcome, patients received the German version of the “EQ-5D-5L” questionnaire from the EuroQol Group during enrollment [35]. Patients were asked to rate their current health-related quality of life between 0 and 100 on a visual analog scale (VAS). After 3 months, the questionnaire was completed again to measure the impact of the interventions on health-related quality of life.

### Sample Size

We performed a priori power analysis using the software G*Power 3.1.9.6 (Heinrich Heine University) which calculates the sample size based on the power, significance level, and effect size [36]. To determine the effect size, we used the study by Sharareh and Schwarzkopf [25] as a baseline, which conducted a group comparison of patient satisfaction with telemedicine. As the resulting effect size represented a very strong effect that we did not expect in our study, we used half of the effect size (1.095) to perform the sample size calculation. This resulted in approximately 19 study participants for each group to achieve a power of 90% in a 2-sided *t* test for independent samples with a global significance level of 5%. The sample size was increased by 10% for both groups to accommodate potential dropouts or withdrawals and by another 10% to counteract a potentially skewed distribution of patient satisfaction. This resulted in a case number of 23 patients per randomization arm. To consider the possible loss of power when using nonparametric methods, the sample size was increased to 30 patients per arm, resulting in a total of 60 enrollments.

### Statistical Analysis

The statistical evaluation of the study included descriptive statistics of the demographic characteristics and parameters collected in the questionnaires. Continuous and ordinal data were reported as mean values, SDs, and medians. Categorical data were presented as absolute and relative frequencies. Differences between the 2 study arms were analyzed using the Mann-Whitney *U* test or Fisher exact test, and effect sizes were reported by Pearson correlation coefficient (*r*) or Cramer V. These nonparametric tests were used because most of the data were not normally distributed, assumptions were not met, or the underlying scale was ordinal. For patient and physician satisfaction, a subgroup analysis based on medical indications was performed. In addition, the Wilcoxon signed-rank test was used to evaluate the longitudinal data of the EQ-5D-5L VAS. Owing to incomplete questionnaires, the reported group size (n) was different for each test. The data were analyzed based on intention-to-treat. The *P* value was set a priori at .05 to test 2-sided significance. The Bonferroni-Holm correction was applied but did not affect the reported results.

To examine the suitability of telemedicine for follow-up appointments in detail and to investigate the type of patients who would use telemedicine, a binary logistic regression was performed. The variable “Which option would you choose for your next appointment?” from one of the patient questionnaires was used as the dependent variable, with the dichotomous outcome “telemedical follow-up” or “standard consultation.” The independent variables added to the model were the categorical parameters “group” with the outcome telemedicine group or control group; “indication” in the form of knee or shoulder; “sex” as male or female; “age” divided into the categories 18 to 40 years, 41 to 60 years, and >60 years; and finally, prior experience with video calls. This exploratory model sought to investigate the factors that influence the decision to use telemedicine when offering video consultations in clinical practice. Bootstrap validation was performed to confirm the validity of the results. A receiver operating characteristic curve was used to assess the accuracy of the model.

## Results

### Overview

The patients were recruited and attended their follow-up appointments between September 2020 and April 2021. The last questionnaires for the second data collection of the EQ-5D-5L were sent in July 2021. For organizational reasons, the number of eligible patients could not be recorded until 2 months after the start of recruitment, resulting in a total of 102 eligible patients.

**Figure 1:**
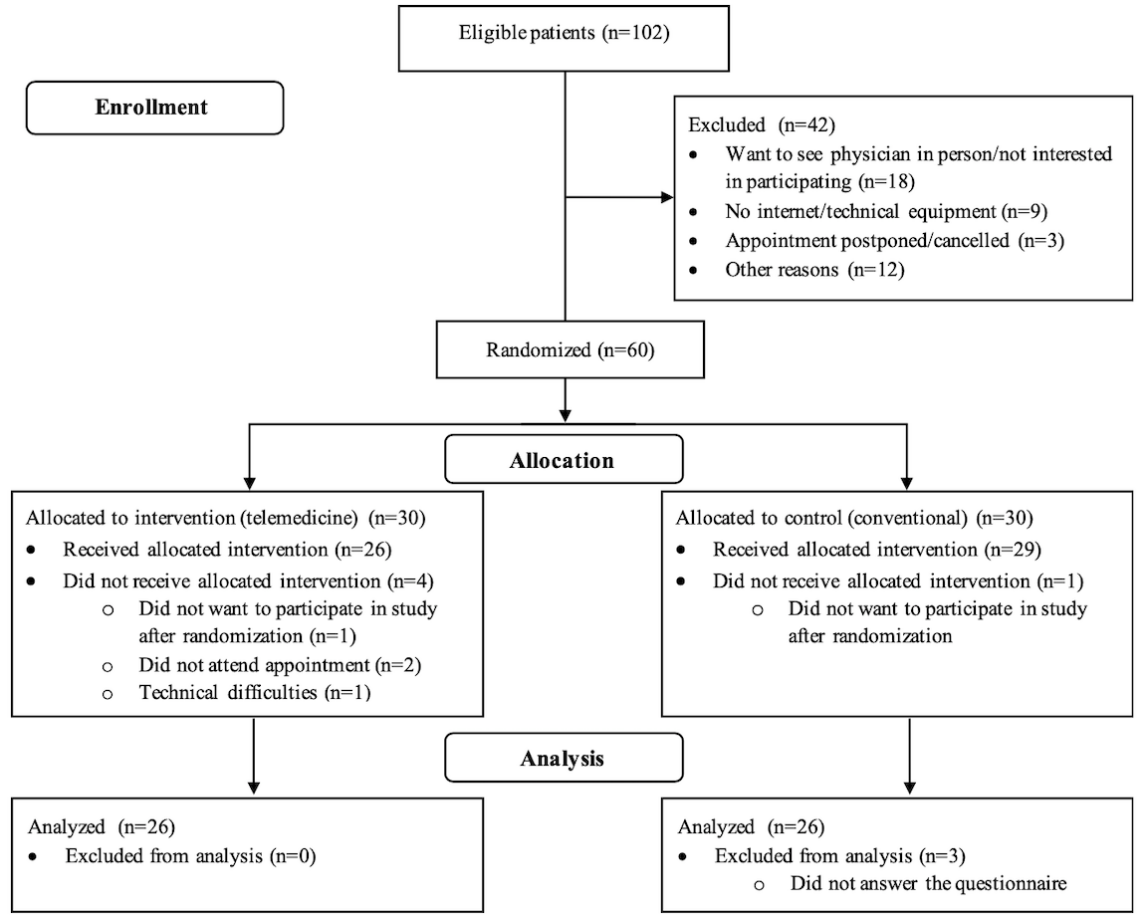
CONSORT (Consolidated Standards of Reporting Trials) flow diagram.

In total, 60 patients agreed to participate in the study and were randomized; 30 patients were allocated to the intervention arm and 30 patients, to the control arm. After randomization, 8 patients withdrew from the study. None of these patients were excluded by the physicians. Thus, 26 patients in the intervention arm and 26 patients in the control arm could be analyzed. Figure 1 shows the CONSORT flow diagram outlining the process of patient recruitment and data analysis. In total, 100% (26/26) of patients in the intervention arm and 90% (26/29) of patients in the control arm completed the questionnaires after the follow-up appointment. With regard to the physician questionnaires, 100% (26/26) in the telemedicine group and 96% (25/26) in the control group were completed. In the intervention group, 100% (26/26) of the EQ-5D-5L questionnaires were returned at baseline, and 69% (18/26) were returned after 3 months; in the control group, 88% (23/26) of the questionnaires were returned at baseline, and 58% (15/26) were returned after 3 months.

Demographic characteristics of patients, such as sex, age, medical indication, distance from clinic, and health status showed no significant differences between the 2 groups (Table 1).

**Table 1:** Demographic characteristics of patients.

			Telemedicine group (n=26)		Control group (n=26)		*P* value	
**Sex, n (%)**								.99^a^
	Female	11 (42)		10 (39)				
	Male	15 (58)		16 (61)				
**Age (years), n (%)**								.36^a^
	18-40	7 (27)		5 (19)				
	41-60	17 (65)		15 (58)				
	>61	2 (8)		6 (23)				
**Medical indication, n (%)**								.99^a^
	Knee	10 (39)		9 (35)				
	Shoulder	16 (61)		17 (65)				
Distance from clinic (km), mean (SD)			37.00 (32.06)		31.58 (22.62)		.65^b^	
Self-assessed health status, mean (SD)			2.88 (1.033)		2.91 (0.848)		.96^b^	

^a^Fisher exact test.

^b^Mann-Whitney *U* test.

### Patient Satisfaction

To measure perceived patient satisfaction, patients in both groups were asked to rate their satisfaction with their respective follow-up appointment using German school grades.

Although group comparison showed that patients were slightly more satisfied with telemedicine follow-up (mean 1.58, SD 0.643) than with in-person follow-up in the clinic (mean 1.64, SD 0.569), the difference was not statistically significant (*P*=.69; Table 2). This result was not affected by a subgroup analysis of the 2 medical indications, namely knee or shoulder. Analysis of the other aspects of the adapted Zufriedenheit in der ambulanten Versorgung–Qualität aus Patientenperspektive questionnaire, such as organization, information, interaction, and participation, showed no significant differences between the groups, with a few exceptions. The waiting time (*P*<.001), atmosphere (*P*<.001), and punctuality of the appointment (*P*=.002) were more satisfying for patients in the telemedicine group than in the control group, with medium to strong effects (*r*=0.440 to *r*=0.760). Box and whisker plots of all distributions can be found in Multimedia Appendix 2.

A strong effect was also evident in the preference for the next follow-up appointment between the groups, which was analyzed with Fisher exact test (*V*=0.542). While patients in the control group preferred to visit the clinic again (16/25, 64%), almost all patients in the telemedicine group (23/26, 88%) chose telemedicine for their next follow-up appointment (*P*<.001). However, a clear majority (32/51, 63%) of all patients chose a video consultation for their next appointment, whereas only 37% (19/51) chose a standard consultation.

**Table 2:** Patient satisfaction.

		Telemedicine group										Control group							*P* value^a^		Pearson correlation coefficient (*r*)		
			Value, n				Value, mean (SD)		Value, median		Value, n			Value, mean (SD)		Value, median							
**Overall patient satisfaction**					26		1.58^b^ (0.643)		1.5		25			1.64^b^ (0.569)		2.00		.69					0.071
	Satisfaction knee patients			10		1.80^b^ (0.632)		2.00		9			1.89^b^ (0.601)		2.00		.95					0.077	
	Satisfaction shoulder patients			16		1.44^b^ (0.629)		1.00		16			1.50^b^ (0.516)		1.5		.72					0.092	
How satisfied are you with the waiting time?					26		2.88^c^ (0.326)		3.00		25			2.12^c^ (0.881)		2.00		<.001					0.546
How satisfied are you with the atmosphere?					26		2.85^c^ (0.368)		3.00		25			2.08^c^ (0.277)		2.00		<.001					0.760
How punctual was your appointment?					26		2.35^c^ (0.689)		2.00		25			1.60^c^ (0.913)		2.00		.002					0.440

^a^Mann-Whitney *U* test.

^b^German school grades; 1=very good to 6=inadequate.

^c^4-point Likert scale; higher scores=higher satisfaction.

### Physician Satisfaction

Physicians in the control group were significantly more satisfied with the follow-up appointments (mean 1.32, SD 0.557) than those in the telemedicine group (mean 2.42, SD 1.419; *P*=.001; *r*=0.466), as shown in Table 3. The subgroup analysis showed that this difference was also significant for the treatment of shoulder patients (*P*=.006) but not for knee patients (*P*=.08). However, a further group comparison, in which video consultations with technical irregularities were removed, revealed no significant group differences in physician satisfaction (mean 1.47, SD 0.516 and mean 1.32, SD 0.557; *P*=.31). In addition, there were no significant differences in their ability to address all relevant medical questions (telemedicine group: 25/26, 96%; control group: 25/25, 100%; *P*=.99; *V*=0.139).

**Table 3:** Physician satisfaction.

		Telemedicine group				Control group				*P* value^a^		Pearson correlation coefficient (*r*)
		Value, n	Value, mean (SD)	Value, median	Value, n		Value, mean (SD)	Value, median				
**Overall physician satisfaction**		26	2.42^b^ (1.419)	2.00	25		1.32^b^ (0.557)	1.00	.001		0.466	
	Satisfaction knee patients	10	2.30^b^ (1.829)	1.50	10		1.10^b^ (0.316)	1.00	.08		0.449	
	Satisfaction shoulder patients	16	2.50^b^ (1.155)	2.00	15		1.47^b^ (0.640)	1.00	.006		0.492	
Physician satisfaction without technical irregularities		15	1.47^b^ (0.516)	1.00	25		1.32^b^ (0.557)	1.00	.31		0.167	

^a^Mann-Whitney *U* test.

^b^German school grades; 1=very good to 6=inadequate.

**Figure 2:**
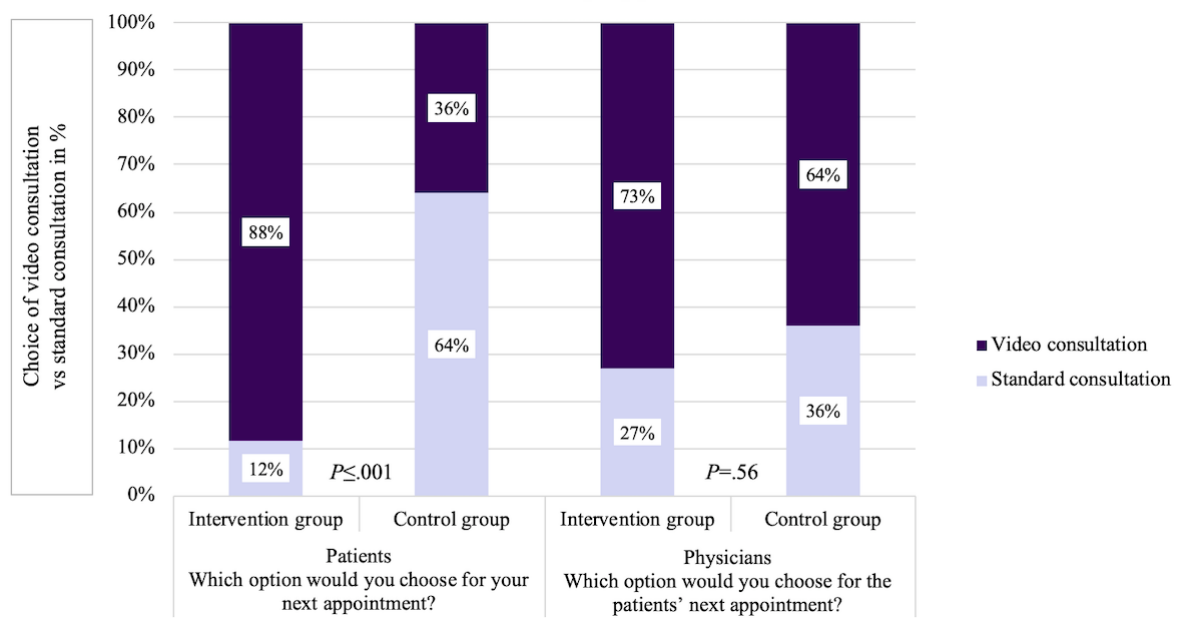
Patient and physician choice of next follow-up appointment.

For the next follow-up appointment, physicians recommended a telemedical consultation for most patients, regardless of the study arm (19/26, 73% of the telemedicine group; 16/25, 64% of the control group; *P*=.56; *V*=0.098). Overall, physicians recommended telemedicine to 69% (35/51) of patients for further follow-up. A comparison of the patients and physicians and their respective choices for the next follow-up appointment is shown in Figure 2.

### Quality of Care

Quality of care was assessed by surveying patients’ perceived health-related quality of life before and after the follow-up visit, using the EQ-5D-5L VAS. As shown in Table 4, the differences in quality of life between the groups were not significant, neither at baseline (*P*=.24) nor after treatment (*P*=.69). The difference in quality of life before and after the follow-up appointment was also not statistically significant between the intervention and control arms (*P*=.19). In this case, the group size changed because only complete data sets could be considered for analysis. In both groups, it was shown with the Wilcoxon signed-rank test that the perceived average quality of life increased after treatment, although not significantly, (telemedicine group: mean 69.77, SD 20.551 to mean 70.44, SD 19.509; *P*=.93; *r*=0.027; control group: mean 66.30, SD 18.292 to mean 69.33, SD 15.216; *P*=.11; *r*=0.440).

**Table 4:** Quality of care.

	Telemedicine group			Control group				*P* value^a^		Pearson correlation coefficient (*r*)
	Value, n	Value, mean (SD)	Value, median	Value, n	Value, mean (SD)	Value, median				
EQ-5D-5L VAS^b^ baseline	26	69.77^c^ (20.551)	79.00	23	66.30^c^ (18.292)	75.00	.24		0.169	
EQ-5D-5L VAS 3 months	18	70.44^c^ (19.509)	72.5	15	69.33^c^ (15.216)	75.00	.69		0.073	
Δ EQ-5D-5L VAS	18	0.833^c^ (16.468)	0.00	14	6.43^c^ (13.921)	6.00	.19		0.237	

^a^Mann-Whitney *U* test.

^b^VAS: visual analog scale.

^c^Scale from 0 to 100.

### Binary Logistic Regression

Binary logistic regression was used to examine the types of patients who would use telemedicine for their follow-up appointment. For this purpose, the potential influence of different variables on patients’ preference for their next follow-up appointment was analyzed (Table 5). The model was statistically significant (*χ^²^*_6_=22.3; *P*=.001; Nagelkerke *R*^2^=48.4%). It was found that medical indication, sex, nor age had a significant influence on the choice of telemedicine. However, previous experience with video calls before the study (*P*=.03) and the respective study arm in which the patients were treated (*P*=.001) contributed significantly to predicting the choice of telemedicine.

**Table 5:** Binary logistic regression.

Variables		Coefficient (β; SE)	*P* value	Odds ratio (95% CI)
Study arm		2.760 (0.836)	.001	15.793 (3.066-81.351)
Indication		.004 (0.810)	.99	1.004 (0.205-4.913)
Sex		−0.686 (0.868)	.43	0.504 (0.092-2.761)
Previous experience with video calls		1.726 (0.802)	.03	5.620 (1.168-27.038)
**Age (years)**				
	18-40	−0.388 (1.280)	.76	0.678 (0.055-8.333)
	41-60	.587 (1.044)	.57	1.799 (0.232-13.919)
	>61 (reference)	—^a^	.59	—
Constant		−1.436 (1.031)	.16	0.238^b^

^a^Reference category.

^b^95% CI value is not applicable.

Patients were 15.8 times more likely to consider telemedicine as a treatment option for further follow-up care if they had already experienced telemedicine than if they had previously been in the control group. Prior experience with general videoconferencing increased the likelihood of participating in telemedicine by 5.6-fold compared with no experience. As a measure of accuracy, the area below the receiver operating characteristic curve was 0.86 (*P*<.001), which indicated that the model has an appropriate fit to predict whether a patient will choose telemedicine for the next follow-up appointment.

## Discussion

### Principal Findings

This study aimed to investigate whether telemedicine can be used efficiently for outpatient orthopedic and trauma surgery follow-up care in Germany in a university hospital from the perspective of patients, physicians, and the quality of care. Our data analysis showed that the use of telemedicine had no significant drawbacks compared with traditional clinical consultations in almost all aspects studied. Patients were even slightly more satisfied with telemedicine, regardless of their medical condition, although the difference was not statistically significant. In addition to overall satisfaction, the authors analyzed specific indicators of satisfaction. Aspects such as waiting time, atmosphere, and punctuality can be significantly improved by using telemedicine. Advantages for patients might arise from the fact that they can interact with their physicians in a familiar environment and do not have to be present in the hospital. The atmosphere, which is the sentiment that patients experience during medical consultations, is perceived to be more pleasant at home than at the hospital. In addition, the waiting time and punctuality of consultations could be evaluated more positively, as patients could use the time at home and are not limited to waiting in the clinic waiting room. In addition, being able to consult a physician from the comfort of home without having to travel and without experiencing long waiting times is a benefit that could have a positive impact on patients’ well-being. The results in the RCTs by Buvik et al [28], Sathiyakumar et al [29], and Kane et al [30] are similar to our findings regarding patient satisfaction. However, these RCTs differ from our study design, as they focus mainly on using telemedicine for individual medical conditions or in a specific setting, such as an outpatient clinic with staff support [28-30].

Although the group comparison showed that physicians were less satisfied with telemedicine than with standard consultations, this lower satisfaction can probably be attributed to technical irregularities. When they were excluded from the analysis, there was no significant difference in satisfaction between the 2 groups for physicians as well. The comparison also showed that technical difficulties have a stronger influence on physician satisfaction than on patient satisfaction. This could be related to the fact that physicians must follow a fixed schedule, which is sensitive to disruptions. The fact that patients are more satisfied with telemedicine than physicians was also identified in the study by Buvik et al [28]. Nevertheless, the high number of video consultations with technical irregularities (11/26, 42%) could have a negative impact on satisfaction with telemedicine over time, and we could not determine whether the irregularities were system related or because of human error. This challenge could be mitigated by the fact that ongoing technological improvements could help make telemedical consultations both easier and more reliable.

Patients’ health-related quality of life did not differ significantly between groups. This might indicate that the application of telemedicine is suitable for the patient group studied and does not have a negative impact on the quality of care. However, it must be noted that telemedicine is suitable only for patients who are already in an advanced stage of the treatment process and who do not currently require follow-up care in the clinic. Therefore, the disease pattern and condition of each patient were reviewed by the physicians before their participation in the study. Generally, the change in quality of life will be less pronounced at this later stage of the treatment process.

All patients in the telemedicine group of this study experienced only 1 telemedical consultation. However, even with this minimal gain in experience, it can be seen that these patients would more frequently opt for telemedicine than those in the control group. Results of other RCTs showed the same conclusion [28-30,37]. Kane et al [30] argued that this could be associated with the fact that people prefer the known rather than the unknown. Thus, familiarity could influence the choice of the type of consultation. However, in addition to this effect, we assume that there is an initial barrier for patients to use telemedicine, for example, technical hurdles. This barrier is overcome for the vast majority of patients once they have participated in their first video consultation. Thereafter, patients were more willing to use telemedicine again, indicating that they considered it an appropriate treatment option. Therefore, it is important to introduce eligible patients to the use of telemedicine and to support them in case of potential uncertainty. At the same time, the learning effect could also increase long-term satisfaction with telemedicine as patients become more confident in using the digital application and learn how to avoid sources of error. Therefore, satisfaction may be even higher among patients who use telemedicine more often. In contrast, physicians recommend telemedicine for most patients, regardless of the study arm. This may be because they prioritize medical value over patients’ prior experiences with this consultation format. The fact that physicians recommend telemedicine despite their initially lower level of satisfaction with it is a further indication of the appropriateness of telemedicine.

Unlike other studies, we also investigated for which patients telemedicine is best suited, as the sensible choice of a promising target group is crucial for the success of telemedicine applications in practice. We were able to show that telemedicine need not be restricted to a specific group of patients but can be provided broadly. Telemedicine was positively evaluated by both knee and shoulder patients with varying ICD-10 codes. Furthermore, binary logistic regression revealed that demographic characteristics had no significant influence on the choice of telemedicine; only prior experience was decisive. This is particularly relevant for clinical practice, as in the long run only the broad-based use of telemedicine for a heterogeneous group of patients is likely to be efficient. When treating single conditions or a small subset of patients, important economies of scale might not be achieved. However, it should be noted that the patients in our trial were comparatively young. Thus, we cannot reject with certainty that a particularly older age might reduce patients’ willingness to participate in telemedicine. However, with the rapid progress of digitalization and its use, this would become less relevant in the future [38].

Nevertheless, the expanded adoption of telemedicine is accompanied by barriers for certain patient groups. In some cases, the use of digital technologies is restricted to older adults, socially deprived people who lack financial means, people without internet access (eg, in rural areas), or members of ethnic minorities [39]. To prevent potential disadvantages and exclusion of these patient groups, policy makers need to consider the following aspects: national internet access infrastructure, access to digital equipment, availability of digital applications in the required languages, deployment of health workers to support patients during video consultations, access to trainings on how to use telemedicine, and the introduction of programs that support digital health literacy [40].

### Limitations

Our study had some limitations. First, we based our sample size calculation on a study by Sharareh and Schwarzkopf [25], which measured a large difference in satisfaction between groups. As a result, our recruited sample size consisted of only 60 patients, which corresponds to a larger expansion of the original sample size calculated. On the basis of our data, we could not detect such a large difference between the groups. Thus, the restricted sample size may have influenced the statistical power of the tests. For example, the results of the binary logistic regression could become more robust with a larger sample size. Furthermore, because of the small sample size, we had to validate the binary logistic regression by bootstrapping and could not split the data set to perform a separate evaluation and validation. Nevertheless, our sample size was comparable with that of other studies, such as that of Kane et al [30].

Another limitation concerns the questionnaires used. International studies have shown that the number of validated questionnaires in this context is limited [23]. This problem is particularly acute in German studies. Therefore, we had to adapt validated questionnaires and partly create them.

The use of pen-and-paper questionnaires, on the one hand, and web-based questionnaires, on the other hand, could also have led to discrepancies. In particular, all questions had to be completed in the web-based questionnaires, but this was not true for the pen-and-paper questionnaires completed in the clinic. However, for organizational reasons, no uniform implementation was possible. This problem also arose for comparable studies. On the other hand, studies have shown that patients usually provide similar health-related answers regardless of survey formats [41-43].

When evaluating the results, it should be noted that all patients recruited from the intervention and control groups consented to participate in telemedicine. Thus, there was an initial interest in telemedicine among participants. This might have led to a self-selection bias in favor of higher satisfaction with telemedicine from the start because patients who were more comfortable with digitalization were more likely to participate in the study [22]. Although all patients consented to undergo a video consultation, it was found that patients in the intervention group were more likely to choose a video consultation for their next follow-up appointment than those in the control group, further supporting the effect of comfortability. Although this self-selection bias is evident for all telemedicine evaluations with a similar study design, it leads to the limitation that the data do not show results for the general population but only show results for patients with a baseline interest in telemedicine [22]. Short of forcing patients to participate in telemedicine, a procedure that appears both unethical in principle and unfeasible in clinical practice, there is no acceptable way of addressing this limitation. In 2018, the percentage of German patients who found video consultations helpful in orthopedic and trauma surgery was 30.5% [31]. However, the higher willingness to participate in our study might suggest that this number will increase in the long term, making our results more generalizable.

Finally, we considered only 1 follow-up consultation in our study design to avoid bias owing to potential learning effects. Thus, no conclusions regarding long-term satisfaction with telemedicine can be made in the context of our study. We suggest that future studies analyze long-term satisfaction with telemedicine in orthopedic and trauma surgery follow-up care. In this context, it should also be investigated how challenges in standard clinic appointments, such as undetected diseases or complications, develop in the context of performing video consultations, particularly in larger patient populations. Future studies concerning the acceptance of telemedicine in Germany and the possible reasons for its rejection would also be of interest. Finally, the causes of technical irregularities should be analyzed in detail to improve the long-term provision of telemedicine.

### Practical Implications

In summary, our results suggest that the effective implementation of telemedical follow-up care ideally meets several conditions. First, the appropriateness of telemedicine should be individually assessed for each patient. In our study, age and sex did not significantly influence telemedicine choice. Nevertheless, physicians should consider whether the patient’s condition and circumstances allow for a video consultation. Before implementing a video consultation in a clinic, criteria should be established to assist with patient selection. These criteria could be based on the inclusion and exclusion criteria of our study, complemented by clinic-dependent characteristics. In addition, suitable patients should be supported to overcome initial uncertainties.

Second, before each consultation, each patient should be assessed individually to determine whether a video consultation is sufficient or whether the patient should attend the clinic. Although physicians in our study would recommend a video consultation as the next appointment for most patients (73% in the intervention group and 64% in the control group), clinical consultations might still be necessary. Moreover, if any medical issues cannot be clarified in a video consultation or if problems occur, additional in-clinic treatment should always be possible, as was the case in this study. To be able to ensure patient safety in the long term, the monitoring, documentation, and control of adverse events is another indispensable factor in this context as well.

Our data refer to patients in orthopedic and trauma surgery. Nevertheless, our results and considerations for practical implications could be transferred to outpatient follow-up examinations in other specialties, such as general and visceral surgeries, if conversations and visual examinations are sufficient for the intended treatment. Therefore, our study could be used as a basis for decision-making regarding the use of telemedicine in different medical fields, supplemented by specialty-specific determinants.

### Conclusions

Compared with international findings, this study highlights that telemedicine is an efficient option for patients in Germany with a broad range of indications in orthopedic and trauma surgery, especially for follow-up appointments. Most patients in the telemedicine group preferred their next follow-up appointment to be a video consultation rather than a standard in-clinic consultation. All patients in this study participated in telemedicine without any prior test run or support from staff, which corresponds to real-life conditions encountered in everyday clinical practice. Clearly, some consultations will always have to occur in hospitals, but telemedicine can be applied efficiently to a wide range of diagnoses and a wide range of patients, thus reducing the burden on patients, physicians, and clinical resources. The COVID-19 pandemic has acted as a catalyst for the widespread uptake of telemedicine. On the one hand, this provided a safe alternative to prevent infections. On the other hand, it demonstrated the benefits of telemedicine. This is why video consultations should find their way into health care beyond the COVID-19 pandemic as a supplement to clinical care.

## Appendix

Multimedia Appendix 1 International Classification of Diseases-10 codes of health conditions studied.

Multimedia Appendix 2 Box and whisker plots.

Multimedia Appendix 3 CONSORT-eHEALTH checklist (V 1.6.1).

## Abbreviations

CONSORT: Consolidated Standards of Reporting Trials
RCT: randomized controlled trial
VAS: visual analog scale
