# Supplementary material for: Assessing Telemedicine Efficiency in Follow-up Care With Video Consultations for Patients in Orthopedic and Trauma Surgery in Germany: Randomized Controlled Trial
Source: J Med Internet Res. 2022 Jul 27;24(7):e36996. doi: 10.2196/36996 (PMC9377439; doi:10.2196/36996)
Supplement: Multimedia Appendix 1 [file jmir_v24i7e36996_app1.docx]

**Multimedia Appendix 1**

| Table S1: International Classification of Disease-10 codes of health conditions studied | |
| --- | --- |
| **Shoulder:**  M75.1, M75.6, M75.0, Z96.60, M75.4, M19.91, S43.1, S42.20, S42.00, M75.2, M75.3, S43.0 | **Knee:**  S83.53, S83.54, S83.2, S83.0, M22.0, M23.32, M23.35, M17.1, M17.5, M21.16, M21.06, S83.3, S83.44, S83.43, S82.18, S82.0, S72.3, S72.43, M25.56, M76.5, S83.6, S76.1, S86.8 |
