# Supplementary material for: Assessing Telemedicine Efficiency in Follow-up Care With Video Consultations for Patients in Orthopedic and Trauma Surgery in Germany: Randomized Controlled Trial
Source: J Med Internet Res. 2022 Jul 27;24(7):e36996. doi: 10.2196/36996 (PMC9377439; doi:10.2196/36996)
Supplement: Multimedia Appendix 2 [file jmir_v24i7e36996_app2.docx]

**Multimedia Appendix 2: Box and whisker plots**


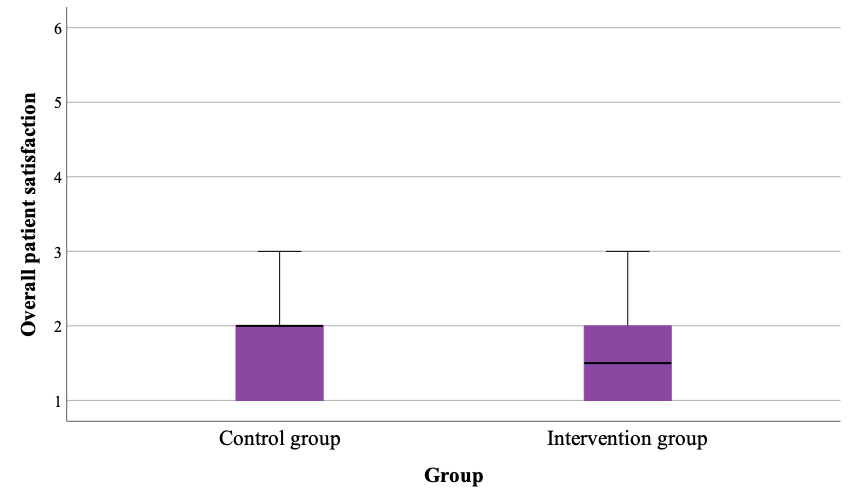


Figure S1: Box and whisker plot patient satisfaction


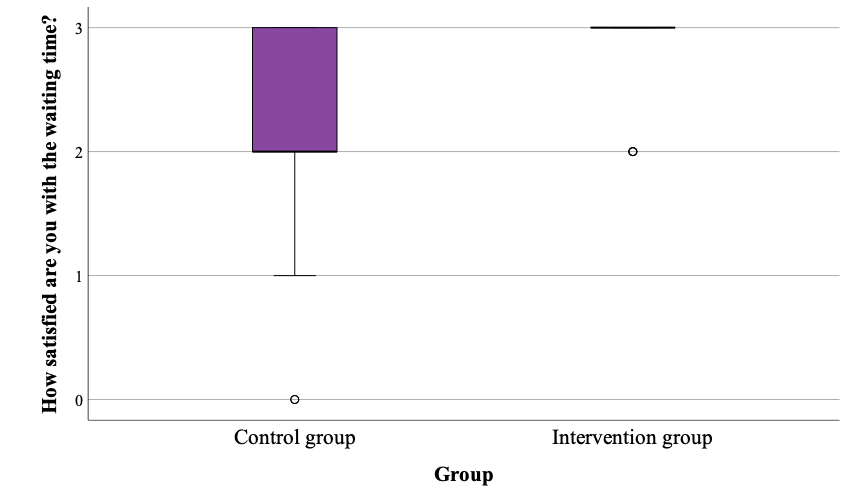


Figure S2: Box and whisker plot waiting time


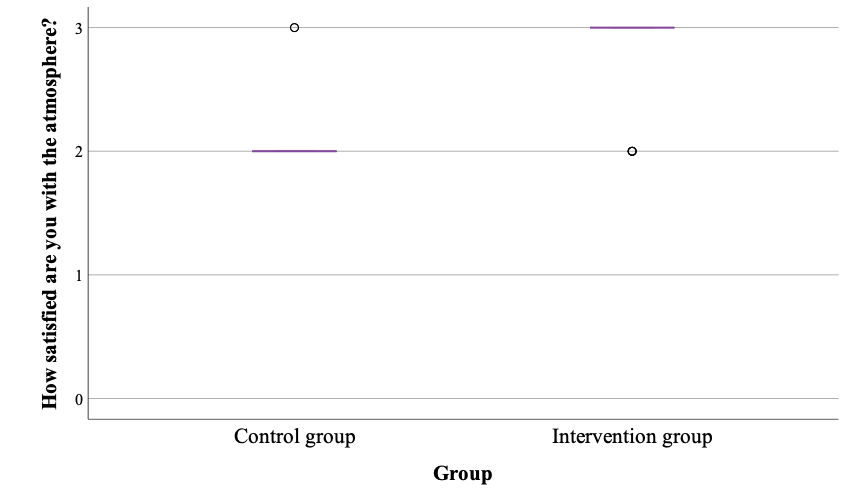


Figure S3: Box and whisker plot atmosphere


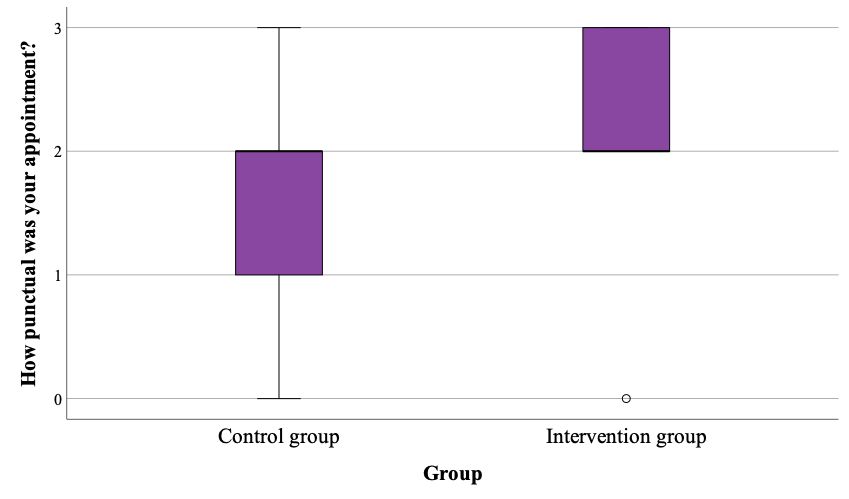


Figure S4: Box and whisker plot punctuality


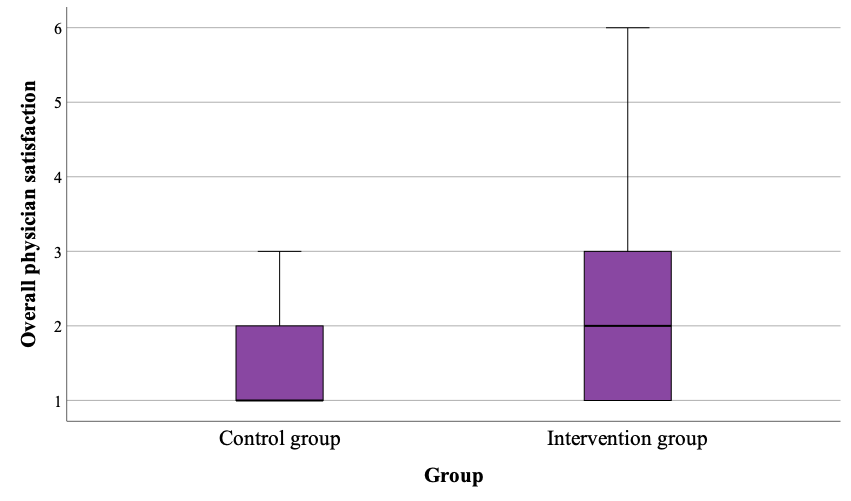


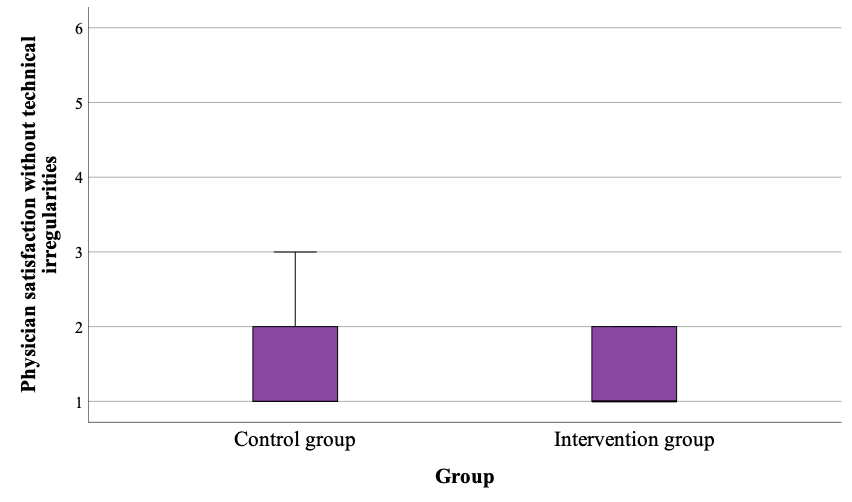
Figure S5: Box and whisker plot physician satisfaction

Figure S6: Box and whisker plot physician satisfaction without technical irregularities


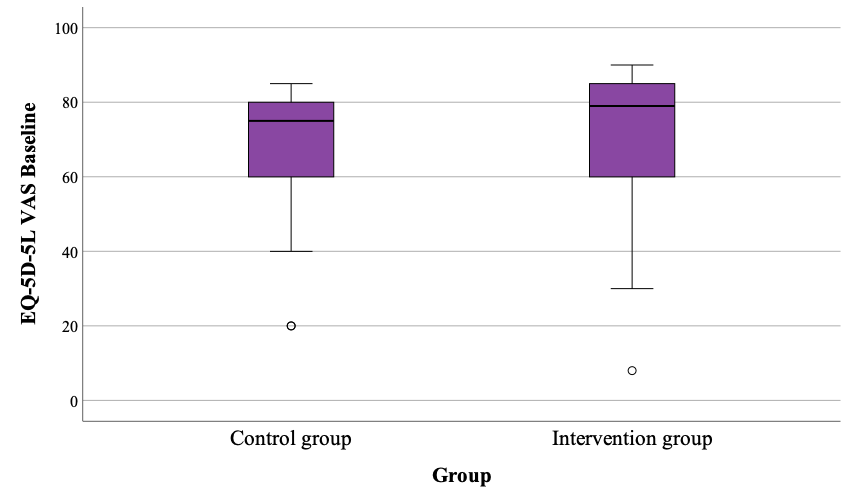


Figure S7: Box and whisker plot EQ-5D-5L VAS baseline


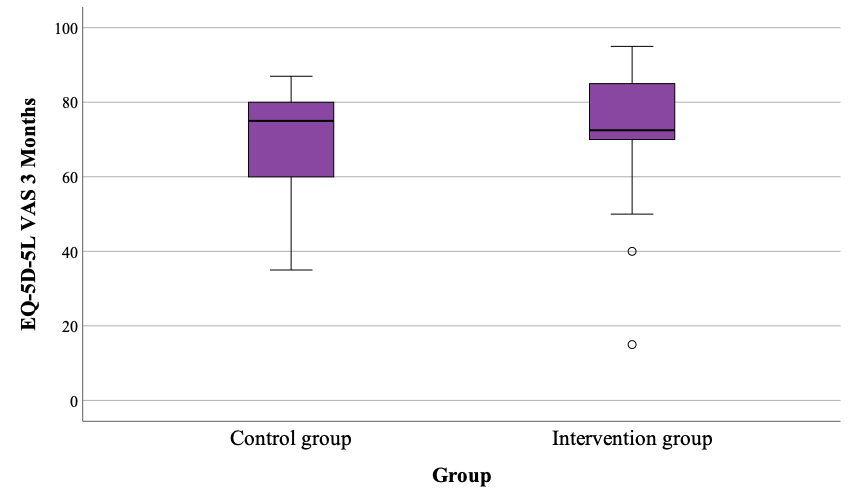


Figure S8: Box and whisker plot EQ-5D-5L VAS 3 months


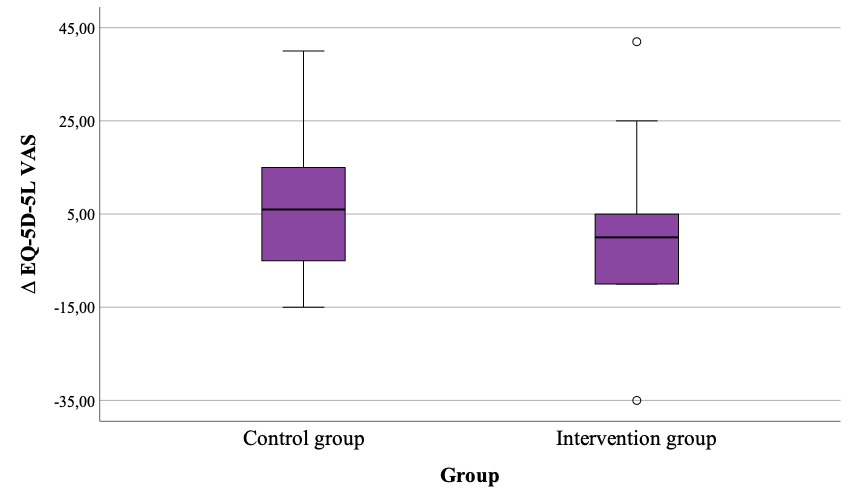


Figure S9: Box and whisker plot ∆ EQ-5D-5L VAS
